# Comparison of BRCA versus non-BRCA germline mutations and associated somatic mutation profiles in patients with unselected breast cancer

**DOI:** 10.18632/aging.102783

**Published:** 2020-02-24

**Authors:** Bo Chen, Guochun Zhang, Xuerui Li, Chongyang Ren, Yulei Wang, Kai Li, Hsiaopei Mok, Li Cao, Lingzhu Wen, Minghan Jia, Cheukfai Li, Liping Guo, Guangnan Wei, Jiali Lin, Yingzi Li, Yuchen Zhang, Han Han-Zhang, Jing Liu, Analyn Lizaso, Ning Liao

**Affiliations:** 1 Department of Breast Cancer, Cancer Center, Guangdong Provincial People's Hospital, Guangdong Academy of Medical Sciences, Guangzhou, Guangdong, China; 2 School of Medicine, South China University of Technology, Guangzhou, China; 3 The Second School of Clinical Medicine, Southern Medical University, Guangzhou, China; 4 Burning Rock Biotech, Guangzhou, China

**Keywords:** germline mutations, BRCA, non-BRCA, somatic mutations, breast cancer

## Abstract

The data on the phenotypes associated with some rare germline mutations in Chinese breast cancer patients are limited. The difference in somatic mutation profiles in breast cancer patients with germline BRCA and non-BRCA mutations remains unexplored. We interrogated the germline and somatic mutational profile of 524 Chinese breast cancer patients with various stages unselected for predisposing factors using a panel consisting of 520 cancer-related genes including 62 cancer susceptibility genes. We divided the patients into three groups according to germline mutations: Germline-BRCA1/2, Germline-others (non-BRCA) and Others (non-carriers). A total of 58 patients (11.1%) carried 76 likely pathogenic or pathogenic (LP/P) germline variants in 15 cancer predisposition genes. Germline *BRCA1/2* mutations were detected from 29 (5.53%) patients; with 11 (2.10%) *BRCA1* carriers and 18 (3.44%) *BRCA2* carriers. In addition, LP/P germline mutations were detected in other genes including *MUTYH* (n=4), *PALB2* (n=4), *ATM* (n=3), *BRIP1* (n=3), *CDH1* (n=3), *RAD51C* (n=3), *CHEK2* (n=2), *FANCA* (n=2), *PMS2* (n=2), *TP53* (n=2), *FANCI* (n=1), *FANCL* (n=1) and *PTEN* (n=1). At least one variant of uncertain significance (VUS) was identified in 490 (93.5%) patients. Young age (P=0.011), premenopausal status (P=0.013), and breast/ovarian cancer family history (P=0.001) were correlated with germline mutations. Germline-BRCA1/2 group was detected with more missense (P=0.02) and less copy-number amplification (P=0.04) than Germline-others group. Meanwhile, Germline-others group and Others group are very similar (P>0.05). The mutation rates of *AKT1*, *CCND1*, *FGFR1*, and *PIK3CA* were different among the three groups. By investigating all breast and ovarian cancer-related genes listed in the US genetic guidelines, we identified 15 cancer susceptibility genes frequently mutated in the germline of our population and must be included in cancer predisposition screening. Our study contributed a better understanding of the tumor characteristics of patients with LP/P germline mutations.

## INTRODUCTION

It is estimated that familial susceptibility to breast cancer accounts for about 25% of all breast cancer cases [1]. The testing for germline mutations in high-penetrance breast cancer predisposition genes has become standard practice for breast cancer patients [2]. In clinical practice, *BRCA1/2* are the most widely tested genes, particularly for breast cancer patients diagnosed at young age, with triple negative breast cancer (TNBC), or have a significant family history of breast, ovarian, or other related cancers [3]. Currently, existing recommendations for germline mutation testing of other high-penetrance genes including *CDH1, TP53* and *PTEN* are based on specific clinical features [4]. Meanwhile, numerous studies have associated mutations in moderate-penetrance genes, including *PALB2*, *ATM*, *CHEK2*, *BRIP1,* with increased breast cancer risk of two to four-fold compared to the 10% risk of the general population [5]. Germline *PALB2* mutations have been reported to play significant roles in hereditary breast cancer, with a five-fold or greater breast cancer risk for mutation carriers [6–8]. In addition, germline mutations in DNA damage repair genes such as *ATM* and *CHEK2* are also associated with an increased risk of breast cancer [9, 10]. Despite mounting evidence suggesting the association of mutations in moderate-penetrance genes with increased breast cancer risk, the current guidelines still do not require the testing of these genes. Thus far, no consensus exists on the number and the specific genes needed to be sequenced and analyzed for the assessment of genetic cancer predisposition [11].

Although harboring germline mutations in either high- or moderate-penetrance genes will increase the predisposition to develop breast cancer, mutations in any one of these genes are rare and testing one gene at a time is both expensive and inefficient [12]. Recent advances in next-generation sequencing (NGS) have made multigene panels more affordable and allowed it to be increasingly used in cancer risk assessment in clinical practice [13, 14]. However, as compared to America and Europe, the use of multigene panel in cancer risk assessment of breast cancer patients is still relatively unpopular in Asia [15]. Expanded multigene panel testing can reveal incidental findings of germline variants in addition to the detection of somatic mutations in highly actionable genes [16]. The detection of likely pathogenic or pathogenic (LP/P) germline variants in low and moderate-risk genes as well as variants of uncertain significance (VUS) also challenges the established genetic counseling repertoire [17, 18]. Moreover, sequencing with multigene panels can identify significant gaps to further understand the relationship between genetics and tumor biology [19].

Numerous studies on germline mutation testing were focused on patients with family history of cancer [20, 21]. Current guidelines require only the patients with known family history to undergo genetic testing; however, not all patients with germline mutations have a known family history of tumors, which results in missing about 50% to 80% of individuals at risk [22, 23]. With growing evidence associating germline mutations with cancer predisposition as well as the availability of targeted therapies, the current view is that all patients newly diagnosed with cancer should be tested for germline mutations, which has the potential to reduce disease burden through secondary prevention and explore targeted therapies [24].

In order to promote the use of multigene panel testing of breast cancer patients, we need to understand the prevalence of germline mutations particularly in cancer predisposition genes beyond *BRCA1/2* to identify the genes commonly mutated in our population. Moreover, the somatic mutation profiles of patients who harbor germline BRCA and non-BRCA mutations remain unexplored. In this study, we interrogated the germline and somatic mutational profile of 524 Chinese breast cancer patients with various stages unselected for predisposing factors, such as age at onset or family history, using a panel consisting of 520 cancer-related genes, including 62 cancer susceptibility genes (Supplementary Table 1). Our study aims to examine the prevalence of germline mutations in known breast cancer predisposition genes and other cancer-associated genes and to evaluate the clinical value of multigene panel testing of germline mutations in this population. We also assessed the relationship between clinicopathologic characteristics and germline mutation status and identified the somatic mutations among germline mutation carriers.

## RESULTS

### Study population

A total of 524 breast cancer patients consented to NGS testing and were offered disclosure of germline results under a separate protocol. Clinical and pathologic features for study patients are provided in Table 1. The mean age at diagnosis was 49.2 years (range, 22 to 86 years). Except for 1 male, all the other 523 patients were females. Most of the patients were diagnosed at stage I (124 cases), II (231 cases) and III (102 cases), while the remaining 67 patients had stage IV. Majority of the patients (82.4%, 432/524) were diagnosed with invasive ductal cancer. Overall, 55 (32.0%) patients reported having a family history of breast or ovarian cancer and 55 (32.0%) patients reported having family history of other cancer. The remaining patients reported no family history of cancer.

**Table 1:** Clinicopathologic features of the study patients.

**Characteristics**	**No.**	**%**
**Age**		
≤40 years	96	18.32%
> 40 years	428	81.68%
**Menopausal status**		
Pre	280	53.44%
Post	231	44.08%
Unknown	12	2.29%
Male	1	0.19%
**Family history of breast or ovarian cancer**		
Yes	93	17.75%
No	330	62.98%
Unknown	101	19.27%
**Tumor size**		
≤2 cm	199	37.98%
> 2 cm	303	57.82%
Unknown	22	4.20%
**Lymph nodes status**		
Negative	256	48.85%
Positive	247	47.14%
Unknown	21	4.01%
**Grade**		
I	23	4.39%
II	234	44.66%
III	234	44.66%
Unknown	33	6.30%
**Histology**		
DCIS	14	2.67%
Infiltrating Ductal Carcinoma	457	87.21%
Infiltrating Lobular	10	1.91%
Carcinoma		
Other, specify	23	4.39%
Unknown	20	3.82%
**ER status**		
Negative	139	26.53%
Positive	363	69.27%
Unknown	22	4.20%

### Frequency and characteristics of deleterious germline mutations

Paired white blood cell and tumor samples from 524 breast cancer patients were sequenced using a panel consisting of 520 cancer-related genes including 62 cancer susceptibility genes to interrogate the germline and somatic mutations, respectively.

**Figure 1:**
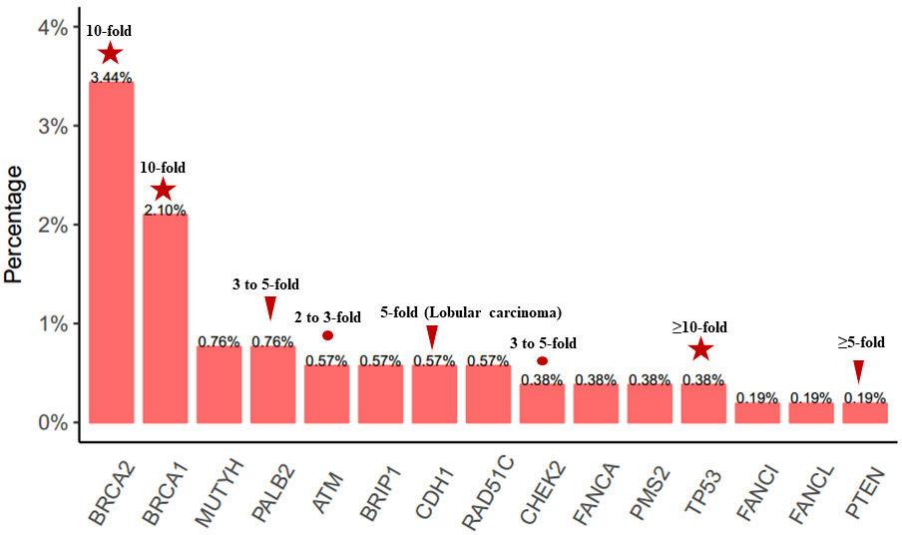
**Frequency and distribution of LP/P germline variants.** LP/P mutations identified in 62 cancer susceptibility genes in 524 unselected breast cancer patients. The multiples of genes associated with breast cancer risk are listed on the histogram.

Analysis revealed the detection of 76 likely pathogenic or pathogenic (LP/P) mutations in 15 cancer susceptibility genes from 58 patients (11.1%, 58/524) (Figure 1). Interestingly, 2 (0.38%) patients had more than one concurrent LP/P germline variants, with *TP53* (c.919+1G>T) and *PMS2* (p.R287fs) mutations detected in a patient with luminal B tumor, and *BRCA2* (p.Q1129*) and *FANCI* (c.158-2A>G) mutations detected in a patient with luminal A tumor.

**Figure 2:**
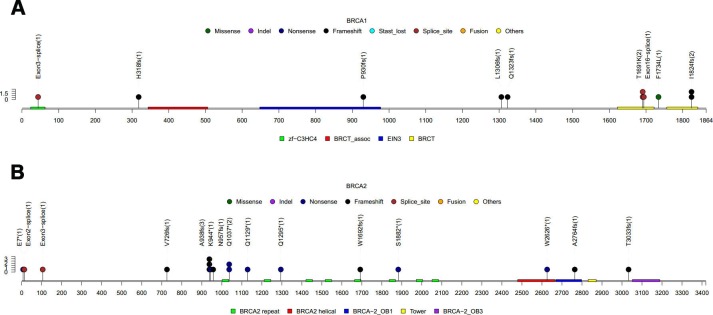
**LP/P germline *BRCA1/2* mutations detected in this cohort.** (**A**) 11 LP/P mutations found in *BRCA1*. (**B**) 18 LP/P mutations found in *BRCA2.* Colored boxes depict the different functional domains along the gene. Small colored circles denote the type of mutation while the location of the circle specifies the mutation site. A patient is represented by a circle. The length of the lollipop represents the number of people of a specific variant.

Twenty-nine (5.53%, 29/524) patients carried germline *BRCA1/2* mutation; including 11 (2.10%, 11/524) patients with *BRCA1* and 18 (3.44%, 18/524) patients with *BRCA2* mutations. As shown in Figure 2A–2B, BRCA1 T1691K (n = 2) and I1824fs (n = 2) and BRCA2 A938fs (n = 3) were the most frequent LP/P germline *BRCA1/2* mutations in our cohort. In addition, 30 (5.72%) patients carried a total of 38 LP/P mutations in other cancer susceptibility genes beyond *BRCA1/2*. Mutations in genes beyond *BRCA1/2* detected in the cohort included *MUTYH* (n = 4), *PALB2* (n = 4), *ATM* (n = 3), *BRIP1* (n = 3), *CDH1* (n = 3), *RAD51C* (n = 3), *CHEK2* (n = 2), *FANCA* (n = 2), *PMS2* (n = 2), *TP53* (n = 2), *FANCI* (n = 1), *FANCL* (n = 1) and *PTEN* (n = 1). Interestingly, among the 15 genes with germline mutations, 7 genes belong to Fanconi anaemia family of genes, including *BRCA2*, *PALB2*, *BRIP1*, *RAD51C*, *FANCA*, *FANCI* and *FANCL*, with 53.4% (31/58) of the patients carrying germline mutations in any of these genes. No LP/P germline mutations were found in the remaining 47 cancer susceptibility genes included in the panel. All the LP/P germline mutations detected in our cohort were listed in Table 2.

**Table 2:** The list of likely pathogenic and pathogenic mutations detected in the cohort.

**Sample ID**	**Gene**	**Mutation type**	**Description**	**AF**	**Degrees**	**Public database or published paper**
RS1829111TIS	ATM	frameshift variant	p.L2081fs	78.90%	Likely pathogenic	N
RSI 829631HS	ATM	stop gained	p.Y155*	43.90%	Likely pathogenic	N
RS1806623TIS	ATM	stop gained	p.E277*	46.82%	Pathogenic	Y (clinvar)
RS1810742TIS	BRCA1	splice region variant	p.T1691K	57.66%	Likely pathogenic	Y (clinvar)
RSI 827051FFP	BRCA1	splice region variant	p.T1691K	72.22%	Likely pathogenic	Y (clinvar)
RS1828137FFP	BRCA1	frameshift variant	p.H318fs	60.59%	Likely pathogenic	Y (PMID: 28724667)
RS1834453TIS	BRCA1	frameshift variant	p.I1824fs	32.62%	Pathogenic	Y (PMID: 28724667)
RS1803594FFP	BRCA1	splice region variant	c.5074+3A>G	58.09%	Pathogenic	Y (clinvar)
RS1726576TIS	BRCAl	missense variant	P-F1734L	80.27%	Likely pathogenic	Y (clinvar)
RS1725206FFP	BRCA1	frameshift variant	p.I1824fs	40.06%	Pathogenic	Y (PMID: 28724667)
RS1827494TIS	BRCAl	frameshift variant	p.P930fs	61.94%	Likely pathogenic	Y (PMID: 28724667)
RS1829599FFP	BRCAl	frameshift variant	p.Q1323fs	64.93%	Pathogenic	N
RSI 811823TIS	BRCAl	frameshift variant	p.L1306fs	84.90%	Pathogenic	Y (PMID: 28724667)
RSI 804518FFP	BRCAl	splice donor variant	c.134+1G>T	48.46%	Pathogenic	Y (clinvar)
RS1815241TIS	BRCA2	frameshift variant	p.A938fs	52.73%	Pathogenic	Y (clinvar)
RS1844984FFP	BRCA2	splice acceptor variant	c.-39-l_-39del	36.90%	Pathogenic	Y (clinvar)
RS1829456TIS	BRCA2	stop gained	p.Q1295*	55.38%	Pathogenic	Y (clinvar)
RS1803222TIS	BRCA2	frameshift variant	p.A2764fs	71.71%	Pathogenic	N
RS1821585FFP	BRCA2	frameshift variant	p.V726fs	51.09%	Likely pathogenic	N
RS1722724TIS	BRCA2	stop gained	p.W2626*	67.38%	Pathogenic	Y (clinvar)
RS1840694PLA	BRCA2	stop gained	p.Q1037*	49.37%	Pathogenic	Y (clinvar)
RSI 823761TIS	BRCA2	stop gained	p.S1882*	82.53%	Pathogenic	Y (clinvar)
RS1833574PLA	BRCA2	stop gained	P-Q1037*	48.98%	Pathogenic	Y (clinvar)
RS1841181FFP	BRCA2	splice donor variant	c.316+lG>A	59.00%	Pathogenic	Y (clinvar)
RS1723884TIS	BRCA2	stop gained	p.K944*	57.95%	Pathogenic	Y (clinvar)
RS1838903TIS	BRCA2	frameshift variant	p.T3033fs	57.11%	Pathogenic	Y (clinvar)
RS1826534FFP	BRCA2	frameshift variant	p.N957fs	57.89%	Pathogenic	N
RS1812099FFP	BRCA2	stop gained	p.Ql 129*	87.60%	Pathogenic	Y (clinvar)
RSI 800551FFP	BRCA2	frameshift variant	p.A938fs	54.00%	Pathogenic	Y (clinvar)
RS1813609FFP	BRCA2	frameshift variant	p.W1692fs	62.10%	Likely pathogenic	Y (PMID: 26689913, PMID: 25415331)
RS1801361TIS	BRCA2	frameshift variant	p.A938fs	56.82%	Pathogenic	Y (clinvar)
RS1813932FFP	BRCA2	stop gained	p.E7*	64.48%	Pathogenic	N
RS1840466TIS	BRIP1	splice donor variant	c.627+lG>A	64.39%	Pathogenic	Y (clinvar)
RS1809229FFP	BRIP1	start lost	p.Ml?	40.17%	Pathogenic	Y (clinvar)
RS1840704PLA	BRIP1	stop gained	P-R798*	48.41%	Pathogenic	Y (clinvar)
RS1828521TIS	CDH1	missense variant	p.T340A	45.90%	Likely pathogenic	Y (clinvar)
RS1829332TIS	CDHl	missense variant	P-T340A	35.80%	Likely pathogenic	Y (clinvar)
RS1830223FFP	CDH1	missense variant	p.T340A	43.68%	Likely pathogenic	Y (clinvar)
RS1726142FFP	CHEK2	stop gained	p.R95*	73.50%	Pathogenic	Y (clinvar)
RSI 829637TIS	CHEK2	missense variant	p.H371Y	78.51%	Likely pathogenic	Y (clinvar)

In addition to the LP/P variants, a total of 1,968 variants of uncertain clinical significance (VUS) in 62 cancer susceptibility genes were also detected in the cohort. At least one VUS was identified in 490 (93.5%) patients, with as many as three variants found per patient. Among them, 53 (10.8%) patients with a VUS also had an LP/P mutation. All the VUSs identified in our cohort are listed in Supplementary Table 2.

### Germline mutations according to breast cancer molecular subtype

We also analyzed the distribution of LP/P germline mutations according to the molecular subtypes of the patients. The distribution and mutation detection rates of the germline mutations detected in our cohort according to their molecular breast cancer subtypes were summarized in Supplementary Tables 3 and 4, respectively.

According to the distribution, germline mutations in the 15 cancer susceptibility genes were found among patients with HR+/HER2- breast tumors (Supplementary Table 3). The overall LP/P germline mutation detection rates were 14.8% (9/61) for patients with triple-negative tumors, 8.5% (5/59) for patients with HER2-enriched tumors, 12.4% (33/267) for patients with HR+/HER2- tumors, 8.5% (7/82) for patients with HR+/HER2+ tumors and 7.3% (4/55) for patients with unknown molecular subtype (Supplementary Table 4). No statistical difference was found for the germline mutation rate according to the molecular subtypes (P=0.4).

### Clinicopathological features of germline mutations carriers

Next, we further analyzed the clinicopathological characteristics of the LP/P germline mutation carriers to understand predisposing factors associated with the germline mutations. Breast cancer was diagnosed at a significantly younger age in germline mutation carriers as compared to non-carriers (median age: 45 vs 50 years, P=0.011, Table 3). Consistent with a younger age of onset, premenopausal women were more likely to carry LP/P germline mutations (P=0.013). Moreover, germline mutation carriers were more likely to have a family history of breast and/or ovarian cancer (P=0.001, Table 3). However, no further significant correlations were found between germline mutation status and other clinicopathologic factors, including tumor size (P=0.561), lymph node status (P=0.731), grade (P=0.420), histology (P=0.973), ER status (P=0.733), PR status (P=0.673), HER2 status (P=0.514) and a triple-negative phenotype (P=0.416). In addition, we found no significant correlation in the clinicopathological characteristics of patients carrying germline mutations in BRCA versus non-BRCA (data not shown).

**Table 3:** Clinicopathological characteristics between germline mutation carriers and non-carriers.

**Characteristics**	**Non-carriers**		**Germline mutation carriers**		**P-value**
**Age**			0.011*
Median (range)	50 (25-86)			45 (22-72)	
<40 years	78	16.74%	18	31.03%	
> 40 years	388	83.26%	40	68.97%	
**Menopausal status**					0.013a*
Pre	238	51.07%	42	72.41%	
Post	216	46.35%	15	25.86%	
Unknown	11	2.36%	1	1.72%	
Male	1	0.21%	0	0.00%	
**Family history of breast or ovarian cancer**				<0.001*
Yes	65	13.95%	28	48.28%	
No	311	66.74%	19	32.76%	
Unknown	90	19.31%	11	18.97%	
**Tumor size**					0.561a
≤2 cm	179	38.41%	20	34.48%	
> 2 cm	266	57.08%	37	63.79%	
Unknown	21	4.51%	1	1.72%	
**Lymph nodes status**					0.731a
Negative	226	48.50%	30	51.72%	
Positive	220	47.21%	27	46.55%	
Unknown	20	4.29%	1	1.72%	
**Grade**					0.420a
I	21	4.51%	2	3.45%	
II	212	45.49%	22	37.93%	
III	202	43.35%	32	55.17%	
Unknown	31	6.65%	2	3.45%	
**Histology**					0.973a
DCIS	13	2.79%	1	1.72%	
Infiltrating Ductal Carcinoma	404	86.70%	53	91.38%	
Infiltrating Lobular Carcinoma	9	1.93%	1	1.72%	
Other, specify	21	4.51%	2	3.45%	
Unknown	19	4.08%	1	1.72%	
**ER status**					0.733a
Negative	124	26.61%	15	25.86%	
Positive	321	68.88%	42	72.41%	
Unknown	21	4.51%	1	1.72%	

### Characteristics of somatic mutations in breast cancer patients with germline mutations

**Figure 3:**
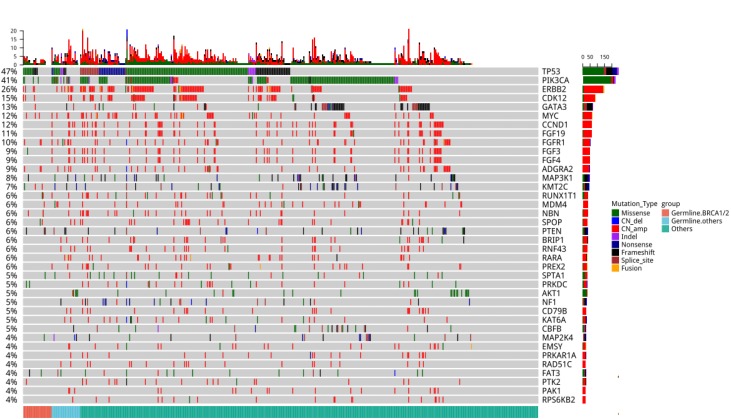
**Comprehensive somatic mutation spectrum of the 524 patients.** Each column represents a patient and each row represents a gene. The number on the left represents the percentage of patients with mutations in a specific gene. The top plot represents the overall number of mutations detected in a patient. Different colors denote different types of mutation. The annotation at the bottom, with each color representing each group, depicts the germline mutations carried by the patients.

To understand the interplay between germline and somatic mutations in breast cancer patients, sequencing data derived from the paired tumor samples of the 524 breast cancer patients were analyzed. Genomic alterations with detection rate of more than 4% were shown in Figure 3. Interestingly, somatic *TP53* mutations were detected in all (100%, 11/11) and a majority (67%, 2/3) of the patients with germline *BRCA1* and *CDH1* mutations, respectively. On the other hand, no somatic *TP53* mutations were detected in all the patients with germline *ATM* (n=3) and *TP53* (n=2). In addition, somatic mutations in *PIK3CA* were more frequent among patients with germline *CDH1* (3/3). Furthermore, a patient with pathogenic germline *PALB2* mutation (p.Q921fs) also had somatic *PALB2* mutation (p.D525fs).

**Figure 4:**
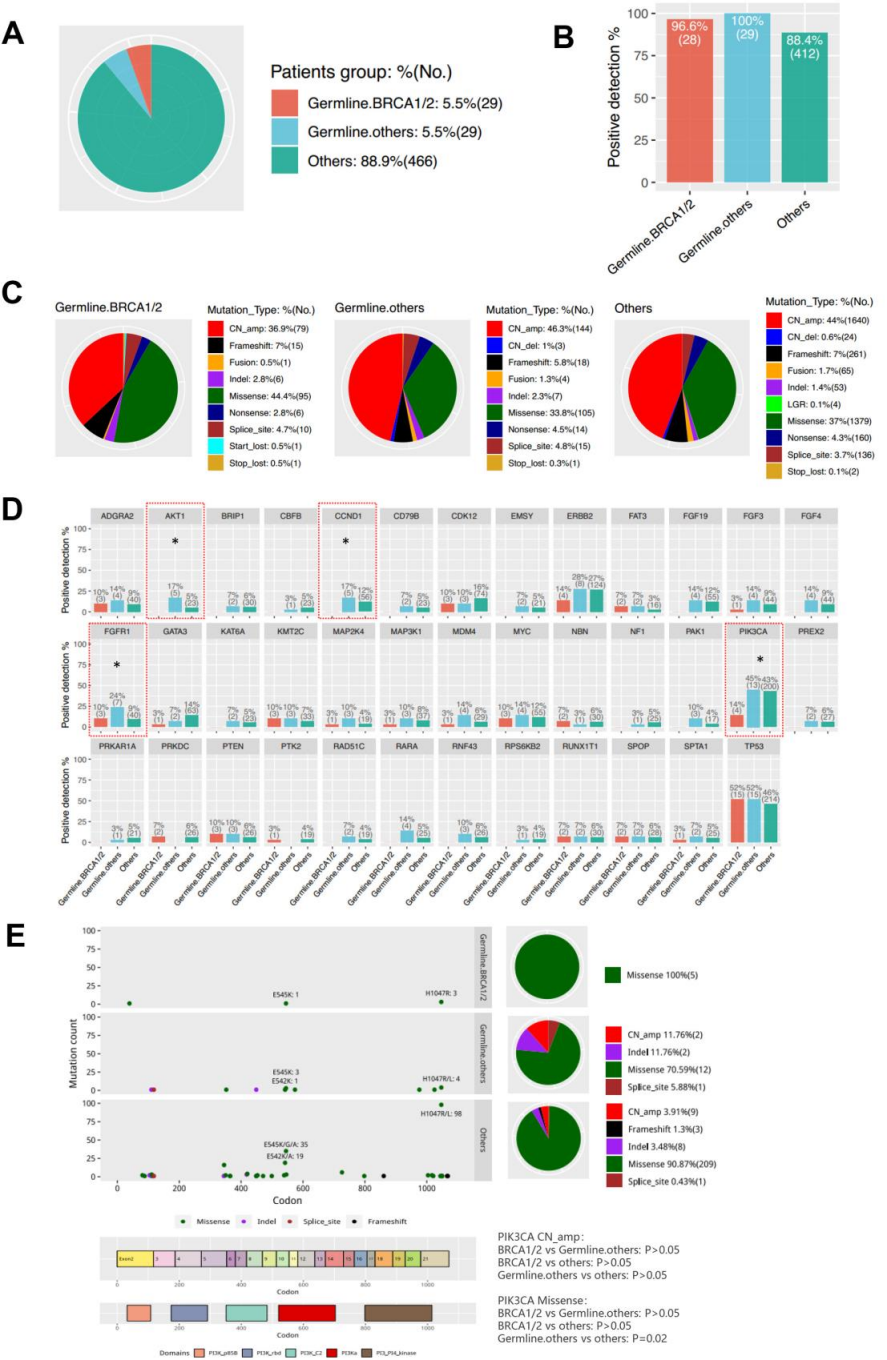
**Characteristics of Somatic Mutations in Breast Cancer Patients with Germline Mutations.** (**A**) Three groups according to germline mutations: Germline-BRCA1/2, Germline-others (non-BRCA) and Others (non-carriers). (**B**) Somatic mutation positive detection rate among the three groups. (**C**) The difference of the mutation type distribution among the three groups. (**D**) The difference of the mutation genes among the three groups. * P<0.05. (**E**). PIK3CA mutation spectrum in the three groups.

Next, we divided the patients into three groups according to germline mutations: Germline-BRCA1/2, Germline-others (non-BRCA) and Others (non-carriers) (Figure 4A). There was no difference in the somatic mutation detection rate among the three groups (Figure 4B). Then, we analyzed the distribution of mutation types among the three groups (Figure 4C). Germline-BRCA1/2 group had significantly more missense mutations (P=0.02) and less copy number amplification (P=0.04) than the Germline-others group. Meanwhile, mutation types between Germline-others group and Others group were not statistically different (P>0.05). Moreover, the mutation rates of *AKT1*, *CCND1*, *FGFR1*, and *PIK3CA* were different among the three groups (Figure 4D and Supplementary Table 5). Mutations in *AKT1* and *CCND1* were not detected in the Germline-BRCA1/2 group. *FGFR1* mutation rate was 24% in Germline-others group, 10% in Germline- BRCA1/2 group, and 9% in Others group. The *PIK3CA* mutation rate was significantly lower in Germline- BRCA1/2 group than the other two groups (Germline-BRCA1/2 vs Germline-others P=0.02; Germline-BRCA1/2 vs Others P=0.002). As shown in Figure 4E, PIK3CA H1047R was the hotspot mutation detected from all three groups. The Others group had significantly more missense *PIK3CA* mutations than the Germline-others group (P=0.02).

## DISCUSSION

In this study, we performed an NGS-based comprehensive analysis of germline mutations in 62 cancer susceptibility genes of 524 unselected Chinese patients with various stages of breast cancer. The inclusion of unselected breast cancer patients with various stages provided a more representative germline mutation landscape among these patients. To our knowledge, by simultaneously interrogating 62 cancer susceptibility genes, our study is the first to elucidate a more comprehensive germline mutation profile of unselected breast cancer patients in the Southern Chinese population.

Our study demonstrated the detection of a total of 76 LP/P germline mutations in 15 genes from 58 patients, revealing an overall germline mutation rate of 11.1%. In contrast, another study of a large cohort of unselected breast cancer patients from North China has revealed an LP/P germline mutation rate of 9.2% (743/8085) [25]. By identifying the 15 genes potentially associated with increased genetic cancer susceptibility in our population, we could advocate for the inclusion of these 15 genes in the routine diagnostic workup for the assessment of genetic cancer predisposition instead of just testing for only *BRCA1/2* mutation status.

A number of studies have reported germline *BRCA1/2* mutation rate of approximately 5% in unselected breast cancer patients regardless of ethnicity [25–30]. Interestingly, we found that Chinese breast cancer patients have more frequent mutations in BRCA2 than in BRCA1 which differ from those in Western breast cancer patients [2]. Meanwhile, most of the BRCA2 mutation carriers (77.8%) in our cohort are HR+/HER2-. Therefore, we should not overlook the clinical value of germline mutation test in both TNBC and non-TNBC patients in China. Our analysis also revealed a mutation rate of 5.72% (30/524) in other cancer predisposition genes beyond *BRCA1/2*. Although the frequency of mutations in each gene is much lower than *BRCA1/2*, the collective mutation rate is more than the mutation rate of *BRCA1/2* in our cohort, providing important data for non-*BRCA1/2* mutations in breast cancer patients in Southern Chinese population. In contrast, the recent survey by Sun et al. revealed a mutation rate of 2.9% (237/8085) and 1% (83/8085) in beyond *BRCA1/2* genes and other cancer susceptibility genes, including mutations in *PALB2* (n=56), *TP53* (n=38), *ATM* (n=31), *RAD51D* (n=31), *RECQL* (n=30) and *CHEK2* (n=27) among the most frequent [25]. Interestingly, germline mutation rate of members of the Fanconi anaemia family of genes (including *BRCA2*, *PALB2*, *BRIP1*, *RAD51C*, *FANCA*, *FANCI* and *FANCL*) was 53.4% (31/58) among the patients with germline mutations in our cohort. These data strongly support the inclusion of not only *BRCA1/2*, but also the Fanconi anaemia genes for the assessment of germline mutations in breast cancer patients. In addition to *BRCA1/2*, growing evidences implicate germline mutations in genes involved in homologous recombination repair pathway such as *PALB2* in increased risk of breast and pancreatic cancer [6, 31] and improved sensitivity to therapeutic agents such as platinum-based chemotherapy and poly-ADP-ribose polymerase (PARP) inhibitors [32, 33]. A number of ongoing clinical trials are investigating the association of germline or somatic mutations in genes involved in homologous recombination repair including *PALB2* and response to different therapeutic agents including chemotherapy in the adjuvant or neoadjuvant setting, PARP inhibitors or checkpoint inhibitors [33].

Among the 62 genes interrogated for germline mutation profiling, no LP/P germline mutations were detected in 47 genes from our cohort, indicating that LP/P germline mutations in these genes are rare in our population. In addition, with the comprehensive germline mutational profiling using 62 cancer susceptibility genes, VUS were detected in more than 90% of the patients. With the increase in the use of NGS in clinical practice, a growing number of VUS are being reported [34]. Further studies are required to understand the function of these variants and their association with the development of disease, particularly in this population.

Furthermore, our study has identified distinct somatic mutations among the carriers of germline mutations and non-carriers. We found that mutation type distribution was different among patients carrying germline mutations in *BRCA1/2* than non-*BRCA1/2*. The mutation rates in *AKT1*, *CCND1*, *FGFR1*, and *PIK3CA* were different among the three groups. Interestingly, a patient with pathogenic germline *PALB2* mutation (p.Q921fs) also had somatic *PALB2* mutation (p.D525fs). The coexistence of LP/P germline and somatic mutations in this patient supports the “second-hit” hypothesis of breast cancer development [8].

There are two limitations in our study. First, all the 524 patients were from a single hospital with most of them from the Guangdong-Hong Kong-Macao Greater Bay Area. Second, no data for treatment responses and survival outcomes were available for analysis. Third, the sample size for certain molecular subtype was very limited. Larger nationwide multicenter studies should be conducted and long-term follow-up is needed to investigate the treatment and survival outcomes in germline mutation carriers.

## CONCLUSIONS

Our findings have potential clinical implications. Firstly, our study is the most comprehensive germline mutation study in unselected breast cancer patients in Southern China interrogating all breast or ovarian cancer-related genes listed in the US genetic guidelines. Secondly, our findings may be useful for selecting the subset of breast cancer patients to receive multigene panel testing. The inclusion of the 15 most common cancer susceptibility genes in cancer genetic predisposition screening is clinically relevant for the Chinese population. Thirdly, we explored the important difference of somatic mutation profiles among BRCA, non-BRCA germline mutations carriers and non-carriers. It provided a basis for better understanding of the tumor characteristics of patients with LP/P germline mutations.

## MATERIALS AND METHODS

### Patient selection

This study was approved by the institutional review board of the Guangdong Provincial People's Hospital, and all participants provided written informed consent. From March 1, 2016 through December 31, 2018, a total of 524 breast cancer patients (AJCC stage Tis to IV) seen at the Department of Breast Cancer in Guangdong Provincial People's Hospital were offered germline sequencing. The disclosure of results was in accordance to an institutional protocol of matched tumor-germline DNA sequencing. Patients were unselected for age or personal and family history of cancers. Clinical and family history data were obtained from medical records. American Society of Clinical Oncology (ASCO) /College of American Pathologists (CAP) guidelines were used to define estrogen receptor (ER), progesterone receptor (PR), and human epidermal growth factor receptor 2 (HER2) positivity. All breast cancers samples were reviewed by breast pathologists. Genetic test results from this analysis were considered research and were not used for clinical decision making.

### Preparation of plasma and tissue samples

Plasma was separated from blood samples collected in EDTA-treated tubes by centrifugation (1,500 x g, 4°C, 10 min). Plasma fractions were transferred into fresh tubes, centrifuged (16,000 x g, 4°C, 10 min) to remove cell debris, aliquoted into fresh tubes, and stored at -80°C until DNA extraction. Breast cancer tissue samples were obtained by biopsy and processed into FFPE cell blocks.

### DNA extraction

Cell-free DNA (cfDNA) and genomic DNA were isolated from plasma and tissue samples using a QIAamp Circulating Nucleic Acid kit or QIAamp DNA FFPE tissue kit, respectively, according to the manufacturer’s standard protocol (Qiagen, Hilden, Germany). Quantification of DNA obtained from plasma and tissue samples was performed using the Qubit dsDNA assay (Life Technologies, Carlsbad, CA, USA).

### NGS library preparation, sequencing and data analysis

DNA was subjected to end repair, phosphorylation and adaptor ligation. Fragments of size 200–400bp were selected by AMPure beads (Agencourt AMPure XP Kit), followed by hybridization with capture probe baits, hybrid selection with magnetic beads and PCR amplification. A bioanalyzer high-sensitivity DNA assay was subsequently performed to assess the quality and size of the fragments. Indexed paired samples were sequenced on Nextseq500 sequencer (Illumina, Inc., USA) with paired-end reads in a Clinical Laboratory Improvement Amendments (CLIA)/CAP-certified laboratory using a panel consisting of 520 cancer-related genes, spanning 1.64 megabases of the human genome (OncoScreen Plus panel, Burning Rock Biotech, Guangzhou, China) [35]. The panel was designed to capture whole exons of 312 genes and critical exons, introns and promoter regions of the remaining 208 genes. The panel also includes 62 cancer susceptibility genes for profiling the germline variants (Supplementary Table 1). The 62 cancer susceptibility genes included in our study was based on the ACMG version 2.0 [36], National Comprehensive Cancer Network (NCCN) Guidelines Genetic/Familial High-Risk Assessment: Breast and Ovarian, Version 2.2017 [37] and Genomics guidelines [38–40]. Sequencing data were analyzed using the Burrows-Wheeler Aligner followed by Genome Analysis Toolkit with an established somatic and germline variant calling pipeline.

### Identification and classification of germline variants

The reported mutations were further confirmed with dbSNP and ClinVar databases. Additionally, other databases such as BRCA Exchange, and Breast Cancer Information Core [41] were searched along with publications archived in PubMed to confirm the assigned class of the mutation, the level of clinical and functional evidence of the mutation and identify novel mutations. Intervar [16], the computational tool for semi-automated variant interpretation, was used to aggregate the variant annotations from multiple databases, prediction tools and publications at a single site. In the absence of clinical data and *in vitro* functional assay, *in silico* predictions using algorithms that assess phylogenetic conservation and the likelihood of severe physiochemical alterations in the protein structure or function were utilized as prediction tools. All genetic annotations and nomenclature were based on GRCh37/hg19 build. The variants were classified according to the American Society of Medical Genetics and Genomics (ACMG) recommendations for standards of interpretation and reporting of sequence variations. The variants were organized into five classes as follows: pathogenic (Class 5), likely pathogenic (Class 4), variants of uncertain significance (Class 3,) likely benign (Class 2) and benign (Class 1) [36]. Without departing from the scope of this study, only pathogenic and likely pathogenic (LP/P) mutations were further analyzed.

### Statistical methods

Patient characteristics and sequencing results were summarized with descriptive statistics, including medians, means, and standard deviations for continuous data. Demographic, clinical, and pathologic characteristics were compared using the Chi-square test or Fisher’s exact test (categorical variables), as applicable. P<0.05 was considered statistically significant.

### Ethics approval

Primary tumor biopsies were obtained using an Institutional Review Board approved protocol, and this study had been approved by the Ethics Committee of Guangdong Provincial People's Hospital. All patients provided written informed consent for translational research.

## Supplementary Material

Supplementary Tables

Supplementary Table 2

Supplementary Table 5

## References

[1] Balmaña J, Díez O, Castiglione M, Group EG, and ESMO Guidelines Working Group. BRCA in breast cancer: ESMO clinical recommendations. Ann Oncol. 2009 (Suppl 4); 20:19–20. 10.1093/annonc/mdp116 19454451

[2] Tung N, Lin NU, Kidd J, Allen BA, Singh N, Wenstrup RJ, Hartman AR, Winer EP, Garber JE. Frequency of Germline Mutations in 25 Cancer Susceptibility Genes in a Sequential Series of Patients With Breast Cancer. J Clin Oncol. 2016; 34:1460–68. 10.1200/JCO.2015.65.0747 26976419 PMC4872307

[3] Khatcheressian JL, Wolff AC, Smith TJ, Grunfeld E, Muss HB, Vogel VG, Halberg F, Somerfield MR, Davidson NE, and American Society of Clinical Oncology. American Society of Clinical Oncology 2006 update of the breast cancer follow-up and management guidelines in the adjuvant setting. J Clin Oncol. 2006; 24:5091–97. 10.1200/JCO.2006.08.8575 17033037

[4] Han MR, Zheng W, Cai Q, Gao YT, Zheng Y, Bolla MK, Michailidou K, Dennis J, Wang Q, Dunning AM, Brennan P, Chen ST, Choi JY, et al. Evaluating genetic variants associated with breast cancer risk in high and moderate-penetrance genes in Asians. Carcinogenesis. 2017; 38:511–18. 10.1093/carcin/bgx010 28419251 PMC5963497

[5] Hollestelle A, Wasielewski M, Martens JW, Schutte M. Discovering moderate-risk breast cancer susceptibility genes. Curr Opin Genet Dev. 2010; 20:268–76. 10.1016/j.gde.2010.02.009 20346647

[6] Rahman N, Seal S, Thompson D, Kelly P, Renwick A, Elliott A, Reid S, Spanova K, Barfoot R, Chagtai T, Jayatilake H, McGuffog L, Hanks S, et al, and Breast Cancer Susceptibility Collaboration (UK). PALB2, which encodes a BRCA2-interacting protein, is a breast cancer susceptibility gene. Nat Genet. 2007; 39:165–67. 10.1038/ng1959 17200668 PMC2871593

[7] Zhang K, Zhou J, Zhu X, Luo M, Xu C, Yu J, Deng M, Zheng S, Chen Y. Germline mutations of PALB2 gene in a sequential series of Chinese patients with breast cancer. Breast Cancer Res Treat. 2017; 166:865–73. 10.1007/s10549-017-4425-z 28825143

[8] Lee JE, Li N, Rowley SM, Cheasley D, Zethoven M, McInerny S, Gorringe KL, James PA, Campbell IG. Molecular analysis of PALB2-associated breast cancers. J Pathol. 2018; 245:53–60. 10.1002/path.5055 29431189

[9] Reiner AS, Sisti J, John EM, Lynch CF, Brooks JD, Mellemkjær L, Boice JD, Knight JA, Concannon P, Capanu M, Tischkowitz M, Robson M, Liang X, et al, and WECARE Study Collaborative Group. Breast Cancer Family History and Contralateral Breast Cancer Risk in Young Women: An Update From the Women’s Environmental Cancer and Radiation Epidemiology Study. J Clin Oncol. 2018; 36:1513–20. 10.1200/JCO.2017.77.3424 29620998 PMC5959199

[10] Fan Z, Ouyang T, Li J, Wang T, Fan Z, Fan T, Lin B, Xu Y, Xie Y. Identification and analysis of CHEK2 germline mutations in Chinese BRCA1/2-negative breast cancer patients. Breast Cancer Res Treat. 2018; 169:59–67. 10.1007/s10549-018-4673-6 29356917

[11] Taylor A, Brady AF, Frayling IM, Hanson H, Tischkowitz M, Turnbull C, Side L, and UK Cancer Genetics Group (UK-CGG). Consensus for genes to be included on cancer panel tests offered by UK genetics services: guidelines of the UK Cancer Genetics Group. J Med Genet. 2018; 55:372–77. 10.1136/jmedgenet-2017-105188 29661970 PMC5992364

[12] Mandelker D, Zhang L, Kemel Y, Stadler ZK, Joseph V, Zehir A, Pradhan N, Arnold A, Walsh MF, Li Y, Balakrishnan AR, Syed A, Prasad M, et al. Mutation Detection in Patients With Advanced Cancer by Universal Sequencing of Cancer-Related Genes in Tumor and Normal DNA vs Guideline-Based Germline Testing. JAMA. 2017; 318:825–35. 10.1001/jama.2017.11137 28873162 PMC5611881

[13] Slavin TP, Niell-Swiller M, Solomon I, Nehoray B, Rybak C, Blazer KR, Weitzel JN. Clinical Application of Multigene Panels: Challenges of Next-Generation Counseling and Cancer Risk Management. Front Oncol. 2015; 5:208. 10.3389/fonc.2015.00208 26484312 PMC4586434

[14] Bonnet-Serrano F, Bertherat J. Genetics of tumors of the adrenal cortex. Endocr Relat Cancer. 2018; 25:R131–52. 10.1530/ERC-17-0361 29233839

[15] Li JY, Jing R, Wei H, Wang M, Xiaowei Q, Liu H, Jian L, Ou JH, Jiang WH, Tian FG, Sheng Y, Li HY, Xu H, et al. Germline mutations in 40 cancer susceptibility genes among Chinese patients with high hereditary risk breast cancer. Int J Cancer. 2019; 144:281–89. 10.1002/ijc.31601 29752822

[16] Kurian AW, Li Y, Hamilton AS, Ward KC, Hawley ST, Morrow M, McLeod MC, Jagsi R, Katz SJ. Gaps in Incorporating Germline Genetic Testing Into Treatment Decision-Making for Early-Stage Breast Cancer. J Clin Oncol. 2017; 35:2232–39. 10.1200/JCO.2016.71.6480 28402748 PMC5501363

[17] Park KS, Cho EY, Nam SJ, Ki CS, Kim JW. Comparative analysis of BRCA1 and BRCA2 variants of uncertain significance in patients with breast cancer: a multifactorial probability-based model versus ACMG standards and guidelines for interpreting sequence variants. Genet Med. 2016; 18:1250–57. 10.1038/gim.2016.39 27124784

[18] Cobain EF, Milliron KJ, Merajver SD. Updates on breast cancer genetics: clinical implications of detecting syndromes of inherited increased susceptibility to breast cancer. Semin Oncol. 2016; 43:528–35. 10.1053/j.seminoncol.2016.10.001 27899183

[19] Zhang G, Wang Y, Chen B, Guo L, Cao L, Ren C, Wen L, Li K, Jia M, Li C, Mok H, Chen X, Wei G, et al. Characterization of frequently mutated cancer genes in Chinese breast tumors: a comparison of Chinese and TCGA cohorts. Ann Transl Med. 2019; 7:179. 10.21037/atm.2019.04.23 31168460 PMC6526269

[20] Wang J, Li W, Shi Y, Huang Y, Sun T, Tang L, Lu Q, Lei Q, Liao N, Jin F, Li H, Huang T, Qian J, et al. Germline mutation landscape of Chinese patients with familial breast/ovarian cancer in a panel of 22 susceptibility genes. Cancer Med. 2019; 8:2074–84. 10.1002/cam4.2093 30982232 PMC6536923

[21] Thompson ER, Rowley SM, Li N, McInerny S, Devereux L, Wong-Brown MW, Trainer AH, Mitchell G, Scott RJ, James PA, Campbell IG. Panel Testing for Familial Breast Cancer: Calibrating the Tension Between Research and Clinical Care. J Clin Oncol. 2016; 34:1455–59. 10.1200/JCO.2015.63.7454 26786923

[22] Childers CP, Childers KK, Maggard-Gibbons M, Macinko J. National Estimates of Genetic Testing in Women With a History of Breast or Ovarian Cancer. J Clin Oncol. 2017; 35:3800–06. 10.1200/JCO.2017.73.6314 28820644 PMC5707208

[23] Gabai-Kapara E, Lahad A, Kaufman B, Friedman E, Segev S, Renbaum P, Beeri R, Gal M, Grinshpun-Cohen J, Djemal K, Mandell JB, Lee MK, Beller U, et al. Population-based screening for breast and ovarian cancer risk due to BRCA1 and BRCA2. Proc Natl Acad Sci USA. 2014; 111:14205–10. 10.1073/pnas.1415979111 25192939 PMC4191771

[24] Kurian AW, Ward KC, Hamilton AS, Deapen DM, Abrahamse P, Bondarenko I, Li Y, Hawley ST, Morrow M, Jagsi R, Katz SJ. Uptake, Results, and Outcomes of Germline Multiple-Gene Sequencing After Diagnosis of Breast Cancer. JAMA Oncol. 2018; 4:1066–72. 10.1001/jamaoncol.2018.0644 29801090 PMC6143044

[25] Sun J, Meng H, Yao L, Lv M, Bai J, Zhang J, Wang L, Ouyang T, Li J, Wang T, Fan Z, Fan T, Lin B, Xie Y. Germline Mutations in Cancer Susceptibility Genes in a Large Series of Unselected Breast Cancer Patients. Clin Cancer Res. 2017; 23:6113–19. 10.1158/1078-0432.CCR-16-3227 28724667

[26] Koumpis C, Dimitrakakis C, Antsaklis A, Royer R, Zhang S, Narod SA, Kotsopoulos J. Prevalence of BRCA1 and BRCA2 mutations in unselected breast cancer patients from Greece. Hered Cancer Clin Pract. 2011; 9:10. 10.1186/1897-4287-9-10 22085629 PMC3240809

[27] Cancer Genome Atlas N, and Cancer Genome Atlas Network. Comprehensive molecular portraits of human breast tumours. Nature. 2012; 490:61–70. 10.1038/nature11412 23000897 PMC3465532

[28] Abugattas J, Llacuachaqui M, Allende YS, Velásquez AA, Velarde R, Cotrina J, Garcés M, León M, Calderón G, de la Cruz M, Mora P, Royer R, Herzog J, et al. Prevalence of BRCA1 and BRCA2 mutations in unselected breast cancer patients from Peru. Clin Genet. 2015; 88:371–75. 10.1111/cge.12505 25256238 PMC4374018

[29] Wen WX, Allen J, Lai KN, Mariapun S, Hasan SN, Ng PS, Lee DS, Lee SY, Yoon SY, Lim J, Lau SY, Decker B, Pooley K, et al. Inherited mutations in *BRCA1* and *BRCA2* in an unselected multiethnic cohort of Asian patients with breast cancer and healthy controls from Malaysia. J Med Genet. 2018; 55:97–103. 10.1136/jmedgenet-2017-104947 28993434 PMC5800345

[30] Li J, Wen WX, Eklund M, Kvist A, Eriksson M, Christensen HN, Torstensson A, Bajalica-Lagercrantz S, Dunning AM, Decker B, Allen J, Luccarini C, Pooley K, et al. Prevalence of BRCA1 and BRCA2 pathogenic variants in a large, unselected breast cancer cohort. Int J Cancer. 2019; 144:1195–204. 10.1002/ijc.31841 30175445 PMC6320715

[31] Tischkowitz M, Xia B. PALB2/FANCN: recombining cancer and Fanconi anemia. Cancer Res. 2010; 70:7353–59. 10.1158/0008-5472.CAN-10-1012 20858716 PMC2948578

[32] Villarroel MC, Rajeshkumar NV, Garrido-Laguna I, De Jesus-Acosta A, Jones S, Maitra A, Hruban RH, Eshleman JR, Klein A, Laheru D, Donehower R, Hidalgo M. Personalizing cancer treatment in the age of global genomic analyses: PALB2 gene mutations and the response to DNA damaging agents in pancreatic cancer. Mol Cancer Ther. 2011; 10:3–8. 10.1158/1535-7163.MCT-10-0893 21135251 PMC3307340

[33] Isaac D, Karapetyan L, Tamkus D. Association of Germline PALB2 Mutation and Response to Platinum-Based Chemotherapy in Metastatic Breast Cancer: A Case Series. JCO Precis Oncol. 2018; 1–5. 10.1200/PO.17.00258 35135128

[34] Hoffman-Andrews L. The known unknown: the challenges of genetic variants of uncertain significance in clinical practice. J Law Biosci. 2018; 4:648–57. 10.1093/jlb/lsx038 29868193 PMC5965500

[35] Chen B, Zhang G, Wei G, Wang Y, Guo L, Lin J, Li K, Mok H, Cao L, Ren C, Wen L, Jia M, Li C, et al. Heterogeneity of genomic profile in patients with HER2-positive breast cancer. Endocrine-related cancer. 2020; 3:153–162. 10.1530/ERC-19-0414 31905165

[36] Richards S, Aziz N, Bale S, Bick D, Das S, Gastier-Foster J, Grody WW, Hegde M, Lyon E, Spector E, Voelkerding K, Rehm HL; ACMG Laboratory Quality Assurance Committee. Standards and guidelines for the interpretation of sequence variants: a joint consensus recommendation of the American College of Medical Genetics and Genomics and the Association for Molecular Pathology. Genet Med. 2015; 17:405–24. 10.1038/gim.2015.30 25741868 PMC4544753

[37] Daly MB, Pilarski R, Berry M, Buys SS, Farmer M, Friedman S, Garber JE, Kauff ND, Khan S, Klein C, Kohlmann W, Kurian A, Litton JK, et al. NCCN Guidelines Insights: Genetic/Familial High-Risk Assessment: Breast and Ovarian, Version 2.2017. J Natl Compr Canc Netw. 2017; 15:9–20. 10.6004/jnccn.2017.0003 28040716

[38] Rahman N. Realizing the promise of cancer predisposition genes. Nature. 2014; 505:302–08. 10.1038/nature12981 24429628 PMC4975511

[39] Cheng DT, Prasad M, Chekaluk Y, Benayed R, Sadowska J, Zehir A, Syed A, Wang YE, Somar J, Li Y, Yelskaya Z, Wong D, Robson ME, et al. Comprehensive detection of germline variants by MSK-IMPACT, a clinical diagnostic platform for solid tumor molecular oncology and concurrent cancer predisposition testing. BMC Med Genomics. 2017; 10:33. 10.1186/s12920-017-0271-4 28526081 PMC5437632

[40] Gupta S, Provenzale D, Regenbogen SE, Hampel H, Slavin TP Jr, Hall MJ, Llor X, Chung DC, Ahnen DJ, Bray T, Cooper G, Early DS, Ford JM, et al. NCCN Guidelines Insights: Genetic/Familial High-Risk Assessment: Colorectal, Version 3.2017. J Natl Compr Canc Netw. 2017; 15:1465–75. 10.6004/jnccn.2017.0176 29223984

[41] Szabo C, Masiello A, Ryan JF, Brody LC. The breast cancer information core: database design, structure, and scope. Hum Mutat. 2000; 16:123–31. 10.1002/1098-1004(200008)16:2<123::AID-HUMU4>3.0.CO;2-Y 10923033

