# Supplementary material for: Comparison of BRCA versus non-BRCA germline mutations and associated somatic mutation profiles in patients with unselected breast cancer
Source: Aging (Albany NY). 2020 Feb 24;12(4):3140–55. doi: 10.18632/aging.102783 (PMC7066887; doi:10.18632/aging.102783)
Supplement: Supplementary Tables [file aging-12-102783-s003..pdf]

## SUPPLEMENTARY TABLES

**Supplementary Table 1. The list of 62 cancer susceptibility genes included in the gene panel.**

| Major associated tumor types | Gene list |
|---|---|
| Breast and/or ovarian tumors | ATM,BARD1,BRAC1,BRAC2,BRIP1,CDK12,CHEK2,NBN,NF1,PALB2,PPP2R2A,PTEN,RAD51B,STK11,TP53 |
| Gatrointestinal tumors | APC,BMPR1A,CDH1,CHEK1,CHEK2,EPCAM,GREM1,KIT,MLH1,MSH2,MSH3,MSH6,MUTYH,NF1,NTHL1,PDGFRA,PMS2,POLD1,POLE,PPP2R2A,PTEN,SDHA,SDHAF2,SDHB,SDHC,SDHD,SMAD4,STK11,TP53 |
| Melanoma | CDK4,CDKN2A,CHEK1,PTEN |
| Renal tumors | FH,FLCN,MET,PTEN,TP53,VHL,WT1 |
| Hematologic tumors | FANCA,FANCI,FANCL,NBN,PPP2R2A,TP53 |
| Thyroid tumors | MEN1,NF1,PTEN,RET |
| Lung tumors | EGFR,PPP2R2A |
| Prostate cancer | CHEK2,HOXB13,NBN |
| Pancreatic cancer | NBN |
| Schwannoma/Meningioma | NF1,NF2 |
| Carney complex | PRKAR1A |
| Gorlin syndrome | PTCH1 |
| Retinoblastoma | RB1 |
| Familial Paraganglioma syndrome | SDHA,SDHAF2,SDHB,SDHC,SDHD |
| Li-Fraumeni syndrome | TP53 |
| Tuberous Sclerosis | TSC1,TSC2 |
| Wilms Tumor | WT1 |

Please browse Full Text version to see the data of Supplementary Table 2

**Supplementary Table 2. Variants of unknown significance identified in study cohort.**

**Supplementary Table 3. Distribution of LP/P germline mutations according to breast cancer molecular subtype.**

| | Molecular subtype | | | | |
|---|---|---|---|---|---|
| **Cancer susceptibility genes** | **HR-/HER2-** | **HR-/HER2+** | **HR+/HER2-** | **HR+/HER2+** | **Unknown** |
| BRCA1 (n=11) | 4 (36.4%) | 3 (27.3%) | 3 (27.3%) | 1 (9.1%) | 0 (0%) |
| BRCA2 (n=18) | 0 (0%) | 1 (5.6%) | 14 (77.8%)* | 1 (5.6%) | 2 (11.1%) |
| PALB2 (n=4) | 2 (50%) | 0 (0%) | 1 (25%) | 1 (25%) | 0 (0%) |
| MUTYH (n=4) | 1 (25%) | 0 (0%) | 2 (50%) | 1 (25%) | 0 (0%) |
| ATM (n=3) | 0 (0%) | 1 (33.3%) | 1 (33.3%) | 1 (33.3%) | 0 (0%) |
| BRIP1 (n=3) | 0 (0%) | 0 (0%) | 1 (33.3%) | 1 (33.3%) | 1 (33.3%) |
| CDH1 (n=3) | 1 (33.3%) | 0 (0%) | 2 (66.7%) | 0 (0%) | 0 (0%) |
| RAD51C (n=3) | 1 (33.3%) | 0 (0%) | 1 (33.3%) | 0 (0%) | 1 (33.3%) |
| CHEK2 (n=2) | 0 (0%) | 0 (0%) | 2 (100%) | 0 (0%) | 0 (0%) |
| FANCA (n=2) | 0 (0%) | 0 (0%) | 2 (100%) | 0 (0%) | 0 (0%) |
| PMS2 (n=2) | 0 (0%) | 0 (0%) | 1 (50%) | 1 (50%)# | 0 (0%) |
| TP53 (n=2) | 0 (0%) | 0 (0%) | 1 (50%) | 1 (50%)# | 0 (0%) |
| FANCI (n=1) | 0 (0%) | 0 (0%) | 1 (100%)* | 0 (0%) | 0 (0%) |
| FANCL (n=1) | 0 (0%) | 0 (0%) | 1 (100%) | 0 (0%) | 0 (0%) |
| PTEN (n=1) | 0 (0%) | 0 (0%) | 1 (100%) | 0 (0%) | 0 (0%) |

Note: Asterisk (*) denotes the detection of concurrent germline mutation in *BRCA2* and *FANCI* in a patient with HR+/HER2- tumor; Sharp sign (#) denotes the detection of concurrent germline mutation in *TP53* and *PMS2* in a patient with HR+/HER2+ tumor.

**Supplementary Table 4. Germline mutation detection rates according to breast cancer molecular subtype.**

| | Molecular subtype | | | | |
|---|---|---|---|---|---|
| | **HR-/HER2- (n=61)** | **HR-/HER2+ (n=59)** | **HR+/HER2- (n=267)** | **HR+/HER2+ (n=82)** | **Unknown (n=55)** |
| Overall | 9 (14.8%) | 5 (8.5%) | 33 (12.4%) | 7 (8.5%) | 4 (7.3%) |
| BRCA1/2 | 4 (6.6%) | 4 (6.8%) | 17 (6.4%) | 2 (2.4%) | 2 (3.6%) |
| Non-BRCA1/2 | 5 (8.2%) | 1 (1.7%) | 16 (6.0%) | 5 (6.1%) | 2 (3.6%) |

**Supplementary Table 5. The difference of somatic mutations among Germline-BRCA1/2 group, Germline- others group and Others group.**
