# Supplementary material for: Comparison of BRCA versus non-BRCA germline mutations and associated somatic mutation profiles in patients with unselected breast cancer
Source: Aging (Albany NY). 2020 Feb 24;12(4):3140–55. doi: 10.18632/aging.102783 (PMC7066887; doi:10.18632/aging.102783)
Supplement: Supplementary Table 5 [file aging-12-102783-s002..pdf]

Supplementary Table S5. The difference of somatic mutations among Germline-BRCA1/2 group, Germline- others group and Others group

Gene	g_brca.mut	g_others.mut	others.mut	g_brca.wt	g_others.wt	others.wt	p1.g_brca.g_others	p1.g_brca.others	p1.g_others.others	p2.g_brca.g_others	p2.g_brca.others	p2.g_others.others
TP53	15	15	214	14	14	252	p=1	p=0.677	p=0.677	p=1	p=0.57	p=0.57
PIK3CA	4	13	200	25	16	266	p=0.021	p=0.004	p=0.993	p=0.02	p=0.002	p=0.849
ERBB2	4	8	124	25	21	342	p=0.331	p=0.19	p=1	p=0.331	p=0.188	p=1
CDK12	3	3	74	26	26	392	p=1	p=0.593	p=0.593	p=1	p=0.599	p=0.599
GATA3	1	2	63	28	27	403	p=1	p=0.199	p=0.459	p=1	p=0.155	p=0.405
MYC	3	4	55	26	25	411	p=1	p=1	p=0.98	p=1	p=1	p=0.766
CCND1	0	5	56	29	24	410	p=0.061	p=0.093	p=0.59	p=0.052	p=0.062	p=0.384
FGF19	0	4	55	29	25	411	p=0.12	p=0.097	p=0.98	p=0.112	p=0.061	p=0.766
FGFR1	3	7	40	26	22	426	p=0.297	p=1	p=0.014	p=0.297	p=0.731	p=0.014
FGF3	1	4	44	28	25	422	p=0.349	p=0.449	p=0.656	p=0.352	p=0.501	p=0.511
FGF4	0	4	44	29	25	422	p=0.12	p=0.162	p=0.656	p=0.112	p=0.096	p=0.511
ADGRA2	3	4	40	26	25	426	p=1	p=1	p=0.535	p=1	p=0.731	p=0.313
MAP3K1	1	3	37	28	26	429	p=0.604	p=0.602	p=0.912	p=0.611	p=0.716	p=0.721
KMT2C	3	3	33	26	26	433	p=1	p=0.773	p=0.773	p=1	p=0.459	p=0.459
RUNX1T1	2	2	30	27	27	436	p=1	p=1	p=1	p=1	p=0.71	p=0.71
MDM4	1	4	29	28	25	437	p=0.349	p=0.836	p=0.229	p=0.352	p=1	p=0.118
NBN	2	1	30	27	28	436	p=1	p=1	p=0.803	p=1	p=0.71	p=1
SPOP	2	2	28	27	27	438	p=1	p=1	p=1	p=1	p=0.693	p=0.693
PTEN	3	3	26	26	26	440	p=1	p=0.514	p=0.514	p=1	p=0.237	p=0.237
BRIP1	0	2	30	29	27	436	p=0.472	p=0.313	p=1	p=0.491	p=0.244	p=0.71
RNF43	0	3	26	29	26	440	p=0.236	p=0.38	p=0.514	p=0.237	p=0.389	p=0.237
RARA	0	4	25	29	25	441	p=0.12	p=0.399	p=0.142	p=0.112	p=0.387	p=0.081
PREX2	0	2	27	29	27	439	p=0.472	p=0.362	p=1	p=0.491	p=0.392	p=0.684
SPTA1	1	2	25	28	27	441	p=1	p=0.984	p=1	p=1	p=1	p=0.667
PRKDC	2	0	26	27	29	440	p=0.472	p=1	p=0.38	p=0.491	p=0.675	p=0.389
AKT1	0	5	23	29	24	443	p=0.061	p=0.441	p=0.018	p=0.052	p=0.386	p=0.018
NF1	0	1	25	29	28	441	p=1	p=0.399	p=0.984	p=1	p=0.387	p=1
CD79B	0	2	23	29	27	443	p=0.472	p=0.441	p=0.975	p=0.491	p=0.386	p=0.651
KAT6A	0	2	23	29	27	443	p=0.472	p=0.441	p=0.975	p=0.491	p=0.386	p=0.651
CBFB	0	1	23	29	28	443	p=1	p=0.441	p=1	p=1	p=0.386	p=1
MAP2K4	1	3	19	28	26	447	p=0.604	p=1	p=0.261	p=0.611	p=1	p=0.131
EMSY	0	2	21	29	27	445	p=0.472	p=0.488	p=0.89	p=0.491	p=0.626	p=0.637
PRKAR1A	0	1	21	29	28	445	p=1	p=0.488	p=1	p=1	p=0.626	p=1
RAD51C	0	2	19	29	27	447	p=0.472	p=0.541	p=0.798	p=0.491	p=0.618	p=0.352
FAT3	2	2	16	27	27	450	p=1	p=0.649	p=0.649	p=1	p=0.285	p=0.285
PTK2	1	0	19	28	29	447	p=1	p=1	p=0.541	p=1	p=1	p=0.618
PAK1	0	3	17	29	26	449	p=0.236	p=0.602	p=0.197	p=0.237	p=0.614	p=0.105
RPS6KB2	0	1	19	29	28	447	p=1	p=0.541	p=1	p=1	p=0.618	p=1

g_brca=Germline-BRCA1/2； g_others= Germline- others; p1 using chisq.test， p2 using fisher.test；
